# High-Resolution Ribosome Profiling Reveals Gene-Specific Details of UGA Re-Coding in Selenoprotein Biosynthesis

**DOI:** 10.3390/biom12101504

**Published:** 2022-10-17

**Authors:** Simon Bohleber, Noelia Fradejas-Villar, Wenchao Zhao, Uschi Reuter, Ulrich Schweizer

**Affiliations:** 1 Institut für Biochemie und Molekularbiologie, Universitätsklinikum Bonn, Rheinische Friedrich-Wilhelms-Universität Bonn, 53115 Bonn, Germany; 2 Department Medical Biochemistry and Biophysics, Karolinska Institute, SE-171 77 Stockholm, Sweden

**Keywords:** ribosomal footprinting, Ribo-Seq, re-definition, *SECISBP2*, frameshifting

## Abstract

Co-translational incorporation of selenocysteine (Sec) into selenoproteins occurs at UGA codons in a process in which translational elongation competes with translational termination. Selenocysteine insertion sequence-binding protein 2 (*SECISBP2*) greatly enhances Sec incorporation into selenoproteins by interacting with the mRNA, ribosome, and elongation factor Sec (EFSEC). Ribosomal profiling allows to study the process of UGA re-coding in the physiological context of the cell and at the same time for all individual selenoproteins expressed in that cell. Using HAP1 cells expressing a mutant *SECISBP2*, we show here that high-resolution ribosomal profiling can be used to assess read-through efficiency at the UGA in all selenoproteins, including those with Sec close to the C-terminus. Analysis of ribosomes with UGA either at the A-site or the P-site revealed, in a transcript-specific manner, that *SECISBP2* helps to recruit tRNA^Sec^ and stabilize the mRNA. We propose to assess the effect of any perturbation of UGA read-through by determining the proportion of ribosomes carrying UGA in the P-site, pUGA. An additional, new observation is frameshifting that occurred 3′ of the UGA/Sec codon in *SELENOF* and *SELENOW* in *SECISBP2*-mutant HAP1 cells, a finding corroborated by reanalysis of neuron-specific *Secisbp2^R543Q^*-mutant brains.

## 1. Introduction

Several cis- and trans-acting factors are needed to promote the efficient co-translational insertion of selenocysteine (Sec) into selenoproteins directed by UGA/Sec codons [1]. Selenoprotein mRNAs contain a selenocysteine insertion sequence (SECIS), which follows immediately 3′ of the UGA/Sec codon in bacteria or is located in the 3′-untranslated region in eukaryotes [2,3]. In a number of mammalian selenoprotein mRNAs, a selenocysteine redefinition element (SRE) is found in addition to the SECIS element [4,5], and recently, even more sequence determinants within and outside the coding regions of selenoprotein mRNAs have been described [6,7,8]. 

In bacteria, the SECIS element recruits the translation elongation factor SelB, which brings Sec-tRNA^Sec^ to the ribosome [2]. In eukaryotes, these functions are divided between the elongation factor EEFSEC/mSelB [9,10] and the SECIS-binding protein 2 (*SECISBP2*) [11]. The structural details of these interactions have been resolved in greater detail in recent cryo-EM structures of the bacterial ribosome in complex with mRNA, Sec-tRNA^Sec^, and SelB [12] and the mammalian ribosome with mRNA, tRNA^Sec^, EEFSEC, and *SECISBP2* [13]. 

Mutations in *SECISBP2* have been found to impair selenoprotein expression and cause a syndrome of atypical resistance to thyroid hormone [14,15]. Depending on their individual mutations, some of the patients also display additional clinical phenotypes related to neurological, muscular, immune, and hearing impairments [16,17,18]. Frameshift and stop mutations affecting the N-terminus of *SECISBP2* are often not deleterious, because several methionines, including Met300, allow alternative initiation and expression of a functional protein [15]. The minimal functional fragment of *SECISBP2* extends from aa 408–854 (in the human protein), while the function of the N-terminus remains unclear. Pathological mutations affect the Sec incorporation domain (SID, aa 399–517), the lysin-rich domain (aa517–544), and the conserved L7Ae RNA motif (aa 620–745), which interacts with the kink-turn of the SECIS element [19]. 

Mammals contain a paralog of the *SECISBP2* gene, *SECISBP2L* [20]. Mammalian *SECISBP2L* was shown to bind SECIS elements, but a C-terminal fragment containing the RNA binding domain of *SECISBP2L* did not support Sec-incorporation into mammalian selenoproteins [21]. Therefore, the biological significance in vertebrates of *SECISBP2L* remained obscure until studies in zebrafish suggested that few selenoproteins, one tentatively identified as TXNRD1, may utilize *SECISBP2L* for biosynthesis [22]. Very recently, it was shown that the selenoprotein deiodinase 2 may specifically rely on *SECISBP2L* for biosynthesis in developing murine oligodendrocytes [23], but it remains unclear whether *SECISBP2L* supports the biosynthesis of any other mammalian selenoprotein. 

Previously, we studied mice deficient in *Secisbp2* and found to our surprise that complete inactivation of *Secisbp2* in liver or in neurons still allows for some expression of selenoproteins, in particular GPX4 and TXNRD1 [24,25,26]. We modeled two pathogenic missense mutants in a mouse, *SECISBP2*^R543Q^ (located in the lysin-rich domain) and *SECISBP2*^C691R^ (located in the L7Ae domain) [27]. While *SECISBP2*^C691R^ is entirely without function and cannot bind SECIS RNA, *SECISBP2*^R543Q^ is unstable and degraded in liver. However, it is partially functional in neurons and rescues the lethal phenotype of the neuron-specific *Secisbp2* knockout [27]. 

Ribosomal profiling has already changed the study of UGA/Sec re-coding [28]. It allows to study the effects on translational efficiency of all individual selenoproteins without the need of antibodies or activity assays. Total inactivation or mutation of *Secisbp2* in mice was shown to differentially affect UGA/Sec read-through and mRNA abundance [26,27]. However, a major drawback of the in vivo system was the cellular heterogenicity of brain and even liver. Therefore, signals originating from cells not targeted by Cre recombinase expression might have confounded some conclusions about *SECISBP2*-independent selenoprotein expression. In order to address these limitations, we needed a cellular model with three key characteristics: (a) homogeneity, (b) a tolerance for the lack of selenoproteins; and (c) expression of a high number of selenoproteins. In the current study, we used the haploid human myeloid cell line HAP1 harboring a CRISPR-generated *SECISBP2* mutation, which fulfills all of the above characteristics. A second limitation of our previous studies was the incorporation of cycloheximide (CHX) in Ribo-Seq experiments, which arrests ribosomes after selection of a tRNA in the A-site [26,27]. This excluded the opportunity to analyze other informative states of the ribosomes. Therefore, in this study, we subjected the cells to Ribo-Seq without the use of CHX. Consequently, new aspects of UGA re-coding in selenoproteins became accessible to analysis.

## 2. Materials and Methods

Throughout the manuscript, we adhered to the new, systematic nomenclature for selenoproteins [29].

### 2.1. Cell Model Validation and Cell Culture

**Figure 1:**
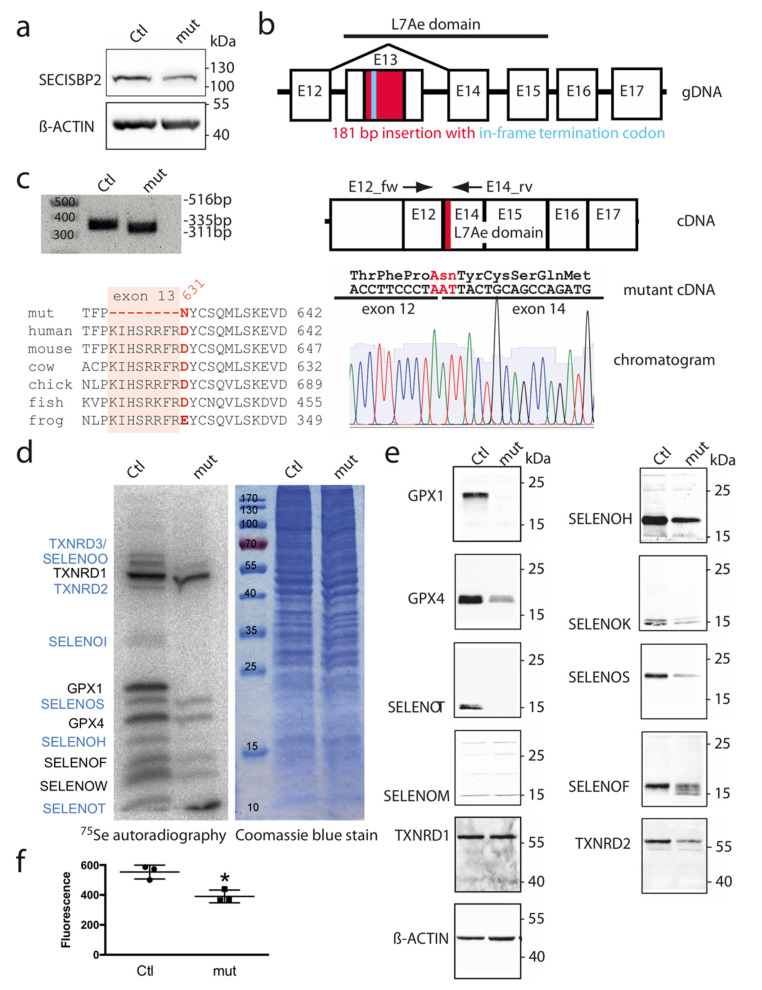
Selenoprotein expression is decreased in *SECISBP2*-mutant HAP1 cells. (**a**) Western blot against *SECISBP2* shows reduced expression of *SECISBP2* in the mutant (mut) cell line compared with the parental cells (Ctl). (**b**) CRISPR-mediated insertion of 181 bp (red) into exon 13 (E13) resulted in an in-frame UGA codon (turquoise) shortly after the insertion site, which was expected to terminate translation at the beginning of the essential L7Ae domain. (**c**) Skipping of exon 13 in *SECISBP2*-mut cells. PCR with primers located in exons 12 and 14 gives the expected product of 335 bp in Ctl cells, but instead of a 516 bp product resulting from the 181 bp insertion, a size of 311 bp is observed. Direct sequencing confirms exon skipping and the Asp631Asn mutation. Alignment of amino acid sequences shows that exon 13 (shaded) is conserved from zebrafish to human. Accession numbers are given in Figure A1. (**d**) Metabolic ^75^Se-labelling of selenoproteins. Autoradiography of SDS-PAGE. Tentative assignments are labeled in blue. Coomassie staining confirms equal loading. Molecular weight markers are indicated. (**e**) Immunoblot analyses of selenoproteins. (**f**) Thioredoxin reductase activity. Cytosolic extract was assayed using the insulin assay. N = 3. * *p* < 0.05 Two-sided Student’s *t* test.

*SECISBP2*-mutant (mut) and the parental HAP1 cell line (Ctl) were purchased from Horizon Discovery (Cambridge, UK). The catalogue numbers are HZGHC003034c012 (RRID: CVCL_TK49) and C631, respectively. HAP1 cells are haploid, thus the CRISPR-mediated insertion of 181 nucleotides into exon 13 of *SECISBP2* modifies all expressed *SECISBP2*. Six codons after the insertion site, a stop codon was expected to terminate translation in the first of three exons comprising the essential L7Ae RNA-binding domain. Sequencing of genomic DNA from *SECISBP2*-mutant HAP1 cells verified the 181 bp genomic insertion (see primers and PCR conditions in Supplementary Material Table S1). Sequencing *SECISBP2* cDNA from the *SECISBP2*-mutant HAP1 cells, however, showed skipping of the mutated exon 13. Exon 12 spliced to exon 14, resulting in an *in-frame* deletion of 8 conserved amino acids (aa 623–630) and mutation of Asp631 to Asn at the splice site (Figure 1c). Primers and PCR conditions are listed in Table S1 in the Supplementary Material Section. The parental cell line was used as control in all experiments. *SECISBP2*-mutant and Ctl cells were grown in Iscove’s Modified Dulbecco’s Medium (IMDM) supplemented with 10% FCS, 100 U/mL Penicillin and 100 μg/mL Streptomycin according to the manufacturer’s instructions. All reagents were purchased from Thermo Fisher Scientific. Cells were cultured at 37 °C in a humidified atmosphere with 5% CO_2_.

### 2.2. Cell Transfection and Luciferase Assay

HAP1 cells grown at the same confluency were transfected using PANFect A (PAN Biotech) with the plasmids described in [30]. After 2 days, the protein was collected using 100 μL of 1× Luciferase cell culture lysis reagent (Promega). Luminescence was measured in triplicates using an Infinite M Plex plate reader (Tecan) by adding 100 μL of luciferase assay reagent (Promega) and 20 μL of the protein lysate. The plate reader was programmed following Promega’s kit recommendations. Luminescence was normalized to protein amount.

### 2.3. Western Blot

Control and *SECISBP2*-mutant HAP1 cells were grown to 80% confluency. The procedure used to detect selenoproteins was previously described [27]. Antibodies used in this study are listed in Table S2. Fusion Solo imaging system (Vilber Lourmat Deutschland GmbH) was used to detect the luminescence produced by horseradish peroxidase (HRP)-conjugated anti-rabbit or anti-mouse antibodies (Jackson Immunotech) and the enhanced HRP chemiluminescence substrate SuperSignal™ West Dura (Thermo Fisher Scientific, Munich, Germany). Equal protein loading was shown by β-actin antibody detection.

### 2.4. [75. Se] Labeling

Control and *SECISBP2*-mutant HAP1 cells were grown to 80% confluency in 60 mm plates (TPP, Switzerland). Cells were labeled with 10 μCi/plate of radioactive sodium selenite (Na_2_[^75^Se]O_3_) overnight. After rinsing with 1× PBS, cells were lysed in RIPA buffer. Fifty micrograms of protein lysate were electrophoresed in a 12% SDS-polyacrylamide gel. Staining of the gel with Coomassie brilliant blue was carried out to show equal loading. The gel was dried using a gel dryer (Bio-Rad) and exposed for one day to a Phosphorimager screen, which was developed by a BAS-1800 II Phosphoimager (Fujifilm), as described in [31].

### 2.5. TXNRD Activity Assay

Assay was performed as in [24] using the insulin-dependent fluorescent TXNRD assay from IMCO (Stockholm, Sweden). The TXNRD activity was calculated as the difference of fluorescence intensity in a time interval within the linear range. Sample background was subtracted. Sample triplicates (three different cell cultures) were used.

### 2.6. Ribosome Profiling and RNA Sequencing

Treatment of the samples was performed as described previously for Ribo-Seq as well as 3′-mRNA sequencing [26,27], with some changes for generating the RPF. Cycloheximide was omitted from the lysis buffer. Ten cm dishes with cells grown to confluency were put on ice and washed once with ice-cold 1× PBS after removal of the media. After addition of 400 μL lysis buffer, the cells were scraped down with a cell scraper. The lysate was transferred to a 1.5 mL reaction tube, and cell clumps were resolved by trituration. The lysate was passed through a 26-gauge needle 10 times. After 10 min incubation on ice, the lysate was centrifuged at 20.000× *g* for 10 min at 4 °C. The supernatant was transferred to a new 1.5 mL reaction tube. Two-hundred μL of the lysate was incubated for 45 min with 1000 U of RNase I. 

### 2.7. Quality Control and Preprocessing of Deep-sequencing Data

The quality of ribosome profiling data was controlled via FastQC v0.11.8 [32] before and after trimming and non-coding RNA removal. Adapter sequences were already removed in the raw data by the manufacturer. Low-quality bases were trimmed using TrimGalore 0.6.0 with cutadapt 2.4 [33,34]. Sequences from ribosome profiling data were first aligned with STAR aligner 2.6.0a [35] (no mismatches allowed) against non-coding RNA sequences obtained from UCSC Genome Browser via the Table Browser tool [36,37]. Unaligned reads (=coding RNA) were then aligned with STAR aligner (with option—*alignEndsType EndToEnd*) against a UCSC hg38 RefSeq transcriptome, containing the longest isoform of corresponding genes with 5′-UTR, CDS and 3′-UTR regions. Results were filtered after unique primary high-quality reads with samtools 1.9 (-F 260 -q 10) for downstream analysis [38]. The size distribution was calculated, and all reads with lengths 20 + 21 nt and 28 + 29 nt were used for further analysis. Offsets in RPFs of these sizes were determined at 12 nucleotides from the 5′ end (first nucleotide in P-site) as previously described [27]. 

3′-mRNA sequencing data were preprocessed using the options recommended by the manufacturer for the QuantSeq 3’-mRNA-Seq Library Prep Kit (Lexogen). Trimmed sequences of the RNA-sequencing data were aligned with STAR aligner 2.6.0a (using the options recommended by the manufacturer) against the GRCh38 human genome retrieved from Ensembl database via biomaRt tool [35,39,40]. Samtools was used to sort and index the resulting files containing the aligned sequences. The number of sequences aligned to transcripts was counted with HTSeq 0.13.5, followed by differential analysis with DESeq2-package v1.14.1 in R 3.3.1 [41,42,43]. For ribosome profiling, reads with lengths of 20 + 21 and 28 + 29 nucleotides, which are located in coding sequences, were used for differential expression analysis. Raw sequence data and raw counts were deposited at the NCBI GEO repository (https://www.ncbi.nlm.nih.gov/geo/), entry GSE145465.

## 3. Results

### 3.1. Deletion of 8 Amino Acids in the L7Ae Domain of SECISBP2 Greatly Reduces Expression of Selenoproteins

**Figure A1:**
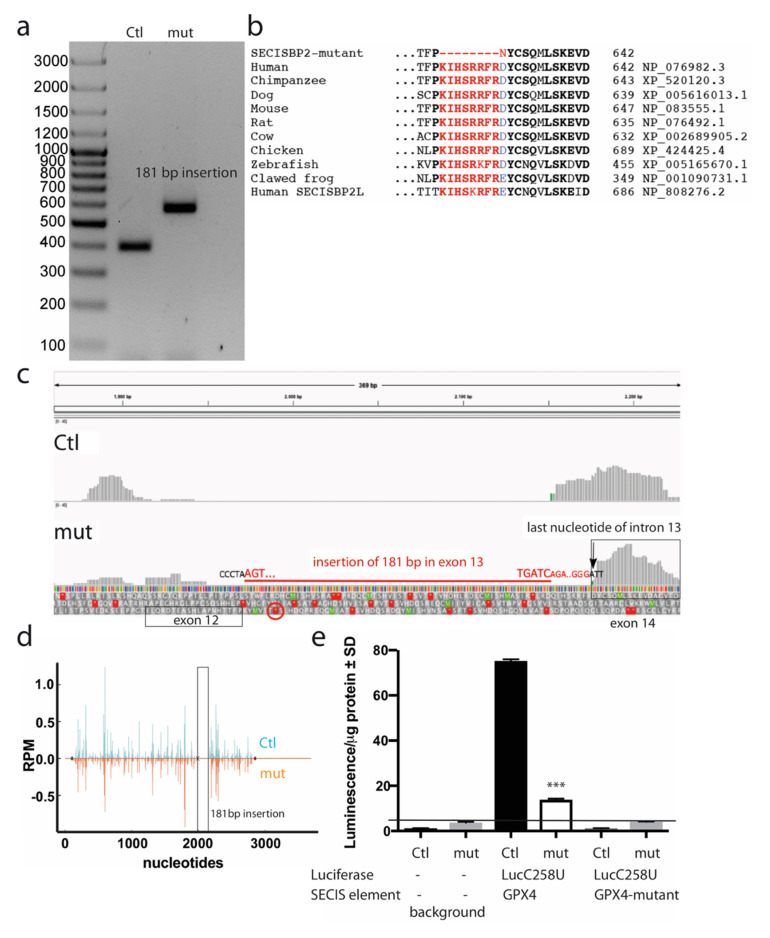
Additional characterization of the mutation in *SECISBP2*-mutant HAP1 cells. (**a**) PCR on genomic DNA confirms the 181 bp insertion in exon 13. Primers used are E12_fw and E14_rv (Table S1). (**b**) Multiple alignment of amino acid sequences around the mutation *SECISBP2^Δ623−630, D631N^* (indicated in red). Accession numbers are given on the right. Asp631 is highly conserved and only in clawed frog conservatively substituted by Glu (blue). The deleted sequence (623–630) and acidic amino acid are highly conserved in the L7Ae domain of *SECISBP2* and *SECISBP2L* (human). (**c**) Integrated Genome Viewer (IGV) view of ribosomal footprints covering the genomic sequence of the mutated *SECISBP2*. The in-frame termination codon is indicated by a circle. Note that there is no ribosomal footprint observed residing on the inserted sequence. (**d**) Translation of *SECISBP2* is intact in *SECISBP2*-mutant cells. Ribosomal coverage of *SECISBP2* over the entire open reading frame appears normal. No decrease is observed after the mutated exon. (**e**) Transient transfection of HAP1 cells with SECIS-dependent luciferase reporter vectors. The construct contains a luciferase open reading frame with Cys258 mutated to Sec followed by a *GPX4*-SECIS element to allow for UGA-recoding [30]. Negative controls include constructs without SECIS or with a SECIS mutated in the essential quartet. *SECISBP2*-mutant (mut) cells have 13% of Ctl luciferase activity when *GPX4* SECIS is included in the vector. N = 3, *** *p* < 0.001, Two-tailed Student’s *t* test.

As a cell model deficient in *SECISBP2*, we used a CRISPR-engineered HAP1 cell line that was expected to produce a truncated *SECISBP2* protein because of an in-frame termination codon in exon 13 (see Methods). However, Western blotting showed decreased amounts of *SECISBP2* protein at the expected molecular weight of 120 kDa as in parental (Ctl) HAP1 cells (Figure 1a). The RNA-binding L7Ae domain of *SECISBP2* is encoded by exons 13-15, and truncation would abrogate all RNA-binding activity (Figure 1b). However, when we amplified the cDNA of *SECISBP2* covering exons 12–14, we found a smaller PCR product instead of a product increased by 181 bp (Figure 1c and Figure A1a). Direct sequencing of the PCR product confirmed that the mutated exon 13 was skipped, resulting in an in-frame fusion of exons 12 and 14. The *SECISBP2*^∆*623−630,D631N*^ mutation comprises deletion of eight highly conserved amino acids and Asp631Asn at the exon junction (Figure 1c and Figure A1b). In ribosomal profiling, we did not observe translation of mutated exon 13 (Figure A1c), and ribosomal coverage of exons 14-17 appeared normal (Figure A1d,e). Therefore, we concluded that the *SECISBP2* mutation produces a *SECISBP2* translation product with a deletion instead of premature termination in the inserted sequence. This deletion plus the non-conservative mutation of amino acid 631 may be the reason for reduced protein abundance observed in Figure 1a. We asked whether this mutant *SECISBP2* protein was functional in the cellular context and transiently transfected Ctl and *SECISBP2*-mutant cells with an established luciferase reporter vector in which translation through a UGA/Sec codon depends on recognition of a GPX4-SECIS element [30]. Activity of the GPX4-SECIS-dependent luciferase reporter was significantly decreased to only 13% of Ctl in *SECISBP2*-mutant cells (Figure A1e). Hence, the mutant *SECISBP2*, while still detectable, must have lost most of its functionality in the cellular context.

Selenoprotein expression was first analyzed by ^75^Se metabolic labeling (Figure 1d). The bands of TXNRD1, GPX1, GPX4, SELENOF, and SELENOW can be inferred from gene-targeting studies. In *SECISBP2*-mutant cells, SELENOI (identified by its mobility), GPX1, and SELENOT (identified by its mobility and response in comparison with Western blotting) were much more strongly decreased than GPX4 and TXNRD1 (Figure 1d). Similar observations were made by Western blotting against selenoproteins (Figure 1e). TXNRD1 carries the Sec in the penultimate position. We therefore measured its activity, which is Sec-dependent, and found a significant decrease in *SECISBP2*-mutant cells (Figure 1f) consistent with its ^75^Se-labeled band intensity (Figure 1d). This observation corresponds to diminished band intensities in other selenoproteins carrying the Sec in a C-terminal position, SELENOS, TXNRD2, and SELENOK (Figure 1e). Taken together, our data clearly show that mutation of *SECISBP2* generally reduced expression of selenoproteins. On the other hand, there was still significant ^75^Se incorporation into several selenoproteins, e.g., GPX4.

### 3.2. Ribosomal Profiling of Selenoprotein Transcripts in HAP1 Cells

**Figure 2:**
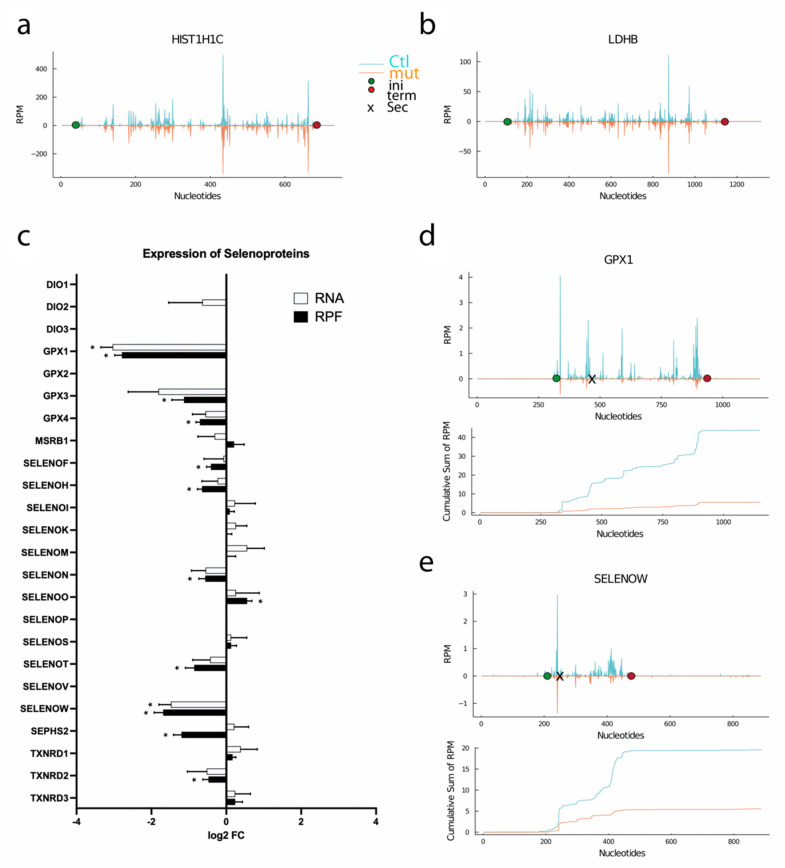
Ribosomal profiling in *SECISBP2*-mutant HAP1 cells. (**a**) Unrelated control gene *HIST1H1C*. (**b**) Unrelated control gene *LDHB*. RPM reads per million mapped reads. (**c**) Comparison of total RNA (from 3′RNA-Seq) and RPF (ribosomal protected fragments) counts in *SECISBP2*-mutant vs. Ctl cells. FC = fold-change. * q < 0.05. Benjamini–Hochberg correction, N = 2. (**d**,**e**) Ribosomal coverage on *GPX1* and *SELENOW* mRNA. The mean values of the genotypes were plotted. The position of the UGA/Sec codon is marked by “**×**”. Start and Stop positions are marked as green and red circles. Cumulative sums of RPF are shown below the corresponding profiles. Ctl, blue; *SECISBP2*-mutant, orange.

In the next experiment, we subjected Ctl and *SECISBP2*-mutant cells to ribosomal profiling and 3′-RNASeq. Two genes, *HIST1H1C* and *LDHB*, were selected as unrelated control genes that should be independent of changes in selenoprotein expression. Both genes show virtually identical ribosomal coverage along their open reading frames in Ctl and *SECISBP2*-mutant cells (Figure 2a,b). We then compared the sum of reads in 3′-RNASeq (RNA) and ribosomal profiling (RPF; ribosome protected fragments) for all selenoproteins (Figure 2c). Deiodinases are not expressed in HAP1 cells, at least they are not being translated. As expected, many genes showed significantly reduced translation, which often correlated closely with reduced mRNA abundance, e.g., *GPX1* and *SELENOW* (Figure 2d,e). These genes are known to be subject to nonsense-mediated decay. 

**Figure 3:**
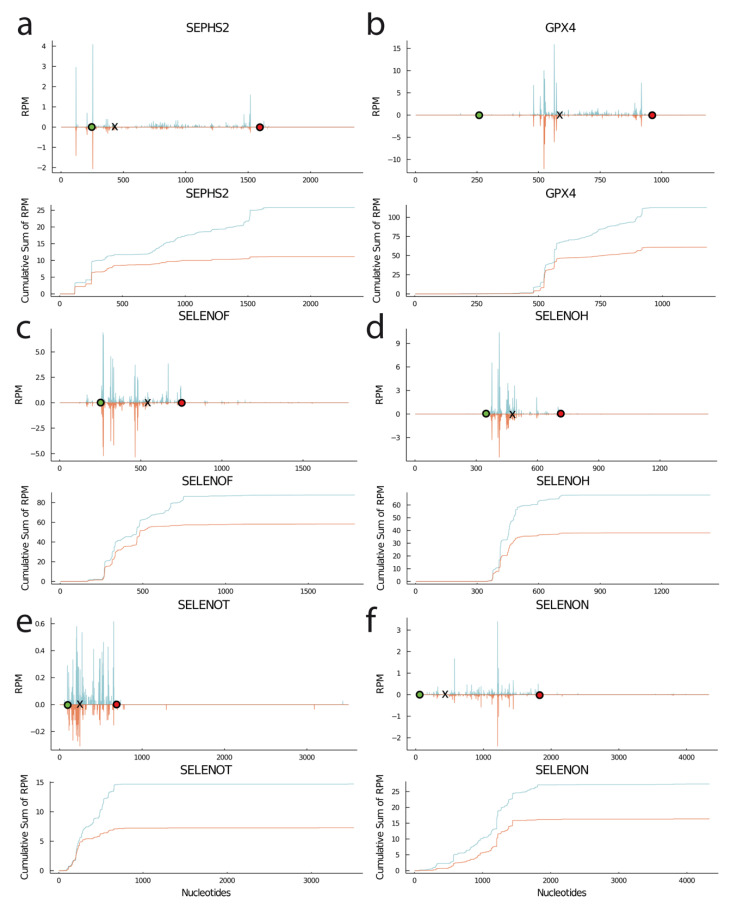
Ribosomal profiling in *SECISBP2*-mutant HAP1 cells. (**a**) *SEPHS2* (**b**) *GPX4* (**c**) *SELENOF* (**d**) *SELENOH* (**e**) *SELENOT* (**f**) *SELENON.* RPM reads per million mapped reads. The mean values of the genotypes were plotted. The position of the UGA/Sec codon is marked by “**×**”. Start and Stop positions are marked as green and red circles. Cumulative sums of RPF are shown below the corresponding profiles. Ctl, blue; *SECISBP2*-mutant, orange.

**Figure A2:**
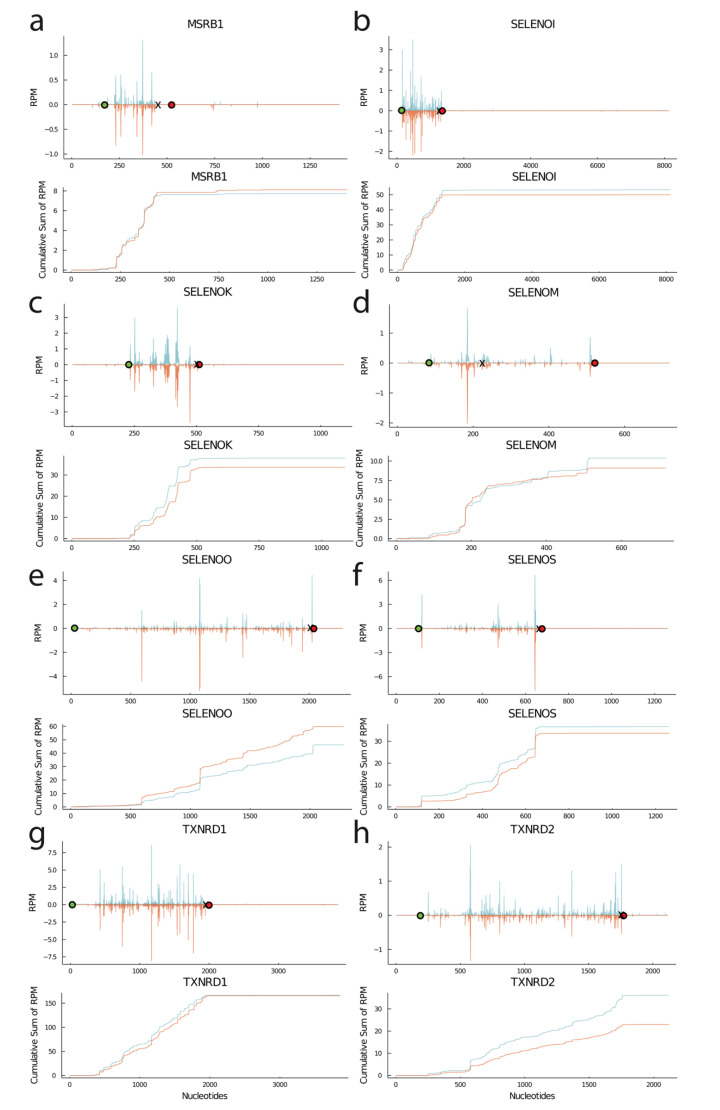
Ribosomal profiling in *SECISBP2*-mutant HAP1 cells. (**a**) *MSRB1,* (**b**) *SELENOI,* (**c**) *SELENOK,* (**d**) *SELENOM,* (**e**) *SELENOO,* (**f**) *SELENOS,* (**g**) *TXNRD1,* and (**h**) *TXNRD2*. The abundance of *TXNRD2* RPFs is, interestingly, reduced despite the UGA/Sec in the penultimate position. RPM reads per million mapped reads. The mean values of the genotypes were plotted. The position of the UGA/Sec codon is marked “×”. Cumulative sums of RPF are shown below the corresponding profiles. Ctl, blue; *SECISBP2*-mutant, orange.

In contrast, *SEPHS2* showed significantly reduced RPF levels in the presence of normal RNA counts (Figure 2c and Figure 3a). Several selenoproteins demonstrated significant changes in RPF abundance dependent on *SECISBP2*, while their RNA abundance was not significantly reduced. These transcripts, *GPX4*, *SELENOH*, *SELENOF*, *SELENON*, *SELENOT*, and *SEPHS2,* are those in which read-through at the UGA is clearly dependent on *SECISBP2* (Figure 3). Other selenoproteins showed little response in either RNA or RPFs to mutation of *SECISBP2*, e.g., *MSRB1*, *SELENOI*, *SELENOK*, *SELENOM*, *SELENOS*, and *TXNRD1* (Figure A2). *SELENOO* seemed even to have increased RPFs (Figure A2). Interestingly, *TXNRD2* shows a significant reduction in RPFs (Figure 2c and Figure A2).

As a rule of thumb, selenoprotein mRNAs that carry the UGA/Sec close to their termination codon are not expected to be sensitive to nonsense-mediated decay (NMD) and, accordingly, mRNA levels and ribosomal coverage should be similar in Ctl and *SECISBP2*-mutant cells. Indeed, this is seen for *MSRB1*, *SELENOI*, *SELENOS,* and *TXNRD1* (Figure A2) but also for *SELENOM,* which has the UGA/Sec codon more centered (Figure A2). In contrast, *SELENOK* and *TXNRD3*, which also have the UGA/Sec close to their termination codon, show reduced amounts of RPF all along the open reading frame (Figure A2). In addition, translation of SELENOS was not decreased, but Western blot nevertheless detected a reduction in protein abundance. We assume that this discrepancy results from targeted degradation of truncated SELENOS protein whose aberrant C-terminus is specifically recognized by a ubiquitin–ligase complex [44,45]. 

### 3.3. Established Readouts of Ribosomal Profiling Underscore the Role of SECISBP2 in UGA Recoding

**Figure 4:**
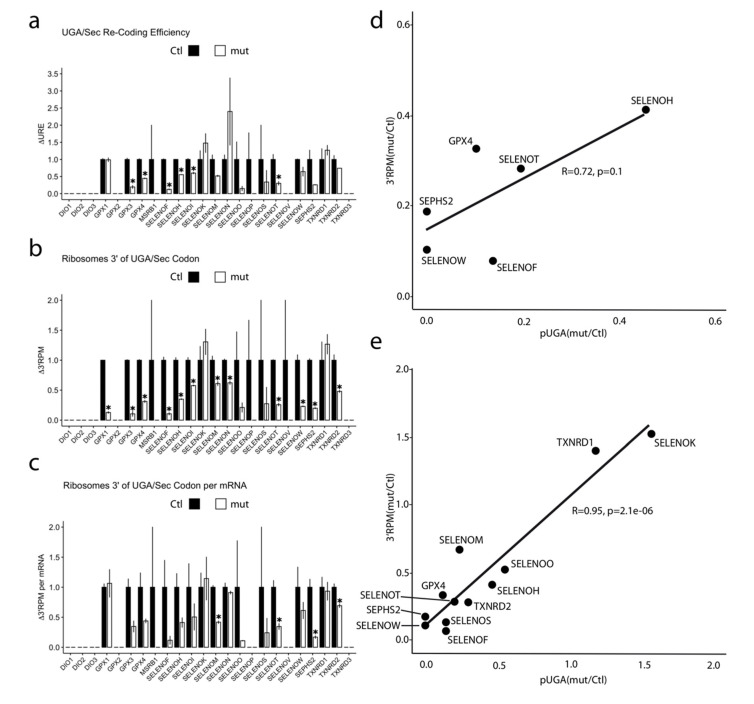
UGA recoding events probed by ribosomal profiling. (**a**) UGA recoding efficiency (URE, 3′RPF/5′RPF) calculated for selenoproteins with UGA/Sec far from the termination codon. URE is calculated as URE(mut)/URE(Ctl). The latter measure is independent of changes in mRNA levels. (**b**) Δ3′RPM (reads 3′ of UGA/Sec per million mapped reads) calculated for selenoproteins. This measure gives a measure for the actual translation of full-length selenoproteins, but it is a function of mRNA abundance. (**c**) Δ3′RPM/mRNA is the same measure as in (**b**) but normalized to mRNA abundance. (**d**) Correlation of 3′RPM with pUGA (UGA in P-site, tRNA selected) for selenoproteins with internal UGA/Sec. (**e**) same correlation including selenoproteins with C-terminal UGA/Sec. The excellent correlation shows that pUGA can be used to approximate UGA/Sec read-through in all selenoproteins. * *p* < 0.05.

Quantitative assessment of UGA/Sec recoding efficiency is not straightforward and still developing. In an attempt to specifically derive a measure of UGA/Sec recoding, we have previously defined URE (UGA redefinition efficiency) ribosomal density 3′ of the UGA/Sec divided by ribosomal density 5′ of the UGA [26,27]. Comparison across models shows that the *SECISBP2*^R543Q^ mutant is less severe than the gene knockout in liver or mutant HAP1 cells (Table A1). We define ΔURE as the quotient of URE of the mutant divided by the URE of the control (Figure 4a). This measure should be independent of prominent spikes in the signals that may result from library preparation artifacts. As expected, ΔURE is reduced for most selenoproteins. The virtues of ΔURE are: (1) the possibility to compare different samples (e.g., mutations with selenium deficiency) and (2) its independence of mRNA abundance. However, it is problematic if another process, i.e., mRNA surveillance, comes into play and eliminates those mRNA species, including the RPFs sitting 5′ of the UGA/Sec (e.g., in *GPX1*). 

A more direct way to assess the final result of selenoprotein translation is thus to quantify the number of RPF 3′ of the UGA/Sec per million mapped reads (3′ RPM). Using this measure, the impairment of selenoprotein translation in *SECISBP2*-mutant cells was evident (Figure 4b). This measure integrates both changes in UGA recoding and in mRNA abundance. Thus, *GPX1* 3′ RPM are significantly reduced. In an attempt to focus on UGA recoding only and eliminate the effect of mRNA degradation, 3′ RPM can be normalized on mRNA abundance (Figure 4c). This measure resembles ΔURE but is not influenced by changes in RPF 5′ of the UGA codon. While all these measures reflect changes in UGA/Sec recoding and are suitable to show the importance of *SECISBP2*, we wondered whether the strengths of Ribo-Seq could be harnessed to reveal even more details of the process.

### 3.4. RPF Containing the UGA/Sec Codon in the Ribosomal A-Site

**Figure 5:**
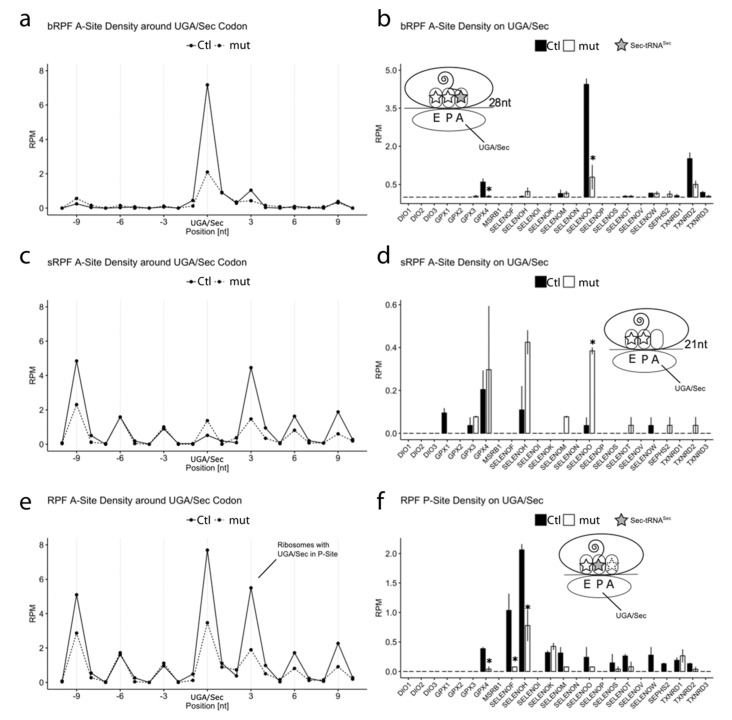
UGA codon occupancy in selenoproteins. RPF are centered on the UGA codon. An offset of 15 nt from the 5′-end indicates that the UGA resides in the A-site. (**a**) Big RPF of 28 + 29 nucleotides length (bRPF, peak at 28 nucleotides) with the UGA/Sec in the A-site expressed as reads per million mapped reads (RPM) over all selenoproteins. (**b**) bRPF with the UGA/Sec in the A-Site expressed as RPM for single selenoproteins. (**c**) Small RPF of 20 + 21 nucleotides length (sRPF, peak 21 nucleotides) with the UGA/Sec in the A-site expressed as RPM over all selenoproteins. (**d**) sRPF with the UGA/Sec in the A-Site expressed as RPM for single selenoproteins. (**e**) RPF of both species combined with the UGA/Sec in the A-site expressed as RPM. (**f**) RPF of both species combined with the UGA/Sec in the P-Site expressed (pUGA) as RPM for single selenoproteins. An offset of 12 nt from the 5′-end to the UGA indicates that the UGA has moved into the P-site (and the A-site occupies the +3 peak in the plot). Read-length distribution is shown in Figure A3. * *p* < 0.05.

The omission of translational inhibitors in our Ribo-Seq experiments allowed us to assess different states of the ribosomes, because ribosomal conformation is reflected by RPF length [46,47]. RPF covering 28 nucleotides (bRPF) are considered to represent a state of the ribosome in which a tRNA occupies the A-site during the peptidyl transfer reaction, while RPF of 21 nucleotides (sRPF) are considered to derive from ribosomes with empty A-sites [47]. In the *SECISBP2*-mutant cells, the fraction of bRPF with UGA in the A-site (i.e., ribosomes having selected a tRNA) is significantly reduced across all selenoproteins (Figure 5a). Depending on sequencing depth, the same can be seen at the level of single selenoproteins. For example, *SELENOO* and *GPX4* clearly show the impairment of UGA/Sec incorporation in *SECISBP2*-mutant cells (Figure 5b), as shown in the coverage plots (Figure 3b and Figure A2).

Conversely, the fraction of sRPF from ribosomes with the UGA/Sec codon in an empty A-site is about two-fold increased across all selenoproteins in the *SECISBP2*-mutant cells (Figure 5c). Since there is no shortage of available selenium (Se) in the culture, this finding likely does not stem from a lack of charged tRNA^Sec^ but may be related to a role of *SECISBP2* in EEFSEC:Sec-tRNA^Sec^ recruitment [13]. This effect is again particularly evident in the case of *SELENOO* (Figure 5d). In *GPX1* there are, however, fewer sRPF covering the UGA in the A-site. This observation might reflect the known susceptibility of *GPX1* to NMD, if *SECISBP2* is lacking. Accordingly, in *GPX4*, the fraction of sRPF with UGA in the A-site does not change, consistent with its mRNA stability. Likewise, the other two mRNAs, which show an accumulation of sRPF on the UGA/Sec (*SELENOH* and *SELENOO*), have stable mRNAs in *SECISBP2*-mutant HAP1 cells.

### 3.5. RPF Containing the UGA/Sec Codon in the Ribosomal P-Site Reflect UGA Re-Definition Efficiency

The probably most direct way to look at the impact of a manipulation on UGA re-definition efficiency is to assess the density of RPF in which the UGA/Sec codon is located in the P-site (pUGA) and relate it to the control situation. In this case, the size of the RPF does not matter, and pooling both RPF sizes leads to larger, more robust numbers. As shown in Figure 5e, pUGA is significantly reduced in *SECISBP2*-mutant cells compared with Ctl. The same can be seen on the level of most individual selenoproteins, consistent with a decreased UGA re-definition efficiency (Figure 5f). pUGA correlates with 3′ RPM in selenoproteins with UGA/Sec far from the C-terminus (R = 0.72, Figure 4d). Including all selenoproteins, i.e., also those with UGA/Sec close to the C-terminus, shows an almost perfect correlation of R = 0.95, supporting pUGA as a suitable measure for UGA re-coding in all selenoproteins (Figure 4e). According to pUGA, UGA re-definition is normal for *TXNRD1*, although its activity is reduced (Figure 1f and Figure 5f). This finding may hint to misincorporation of an amino acid with a related codon [48]. In contrast, pUGA is reduced in *SELENOO*, *SELENOS*, and in *TXNRD2* messages, consistent with their decreased protein abundance (Figure 1d,e and Figure 5f).

### 3.6. The Choices of the Ribosome at a UGA Codon

**Figure 6:**
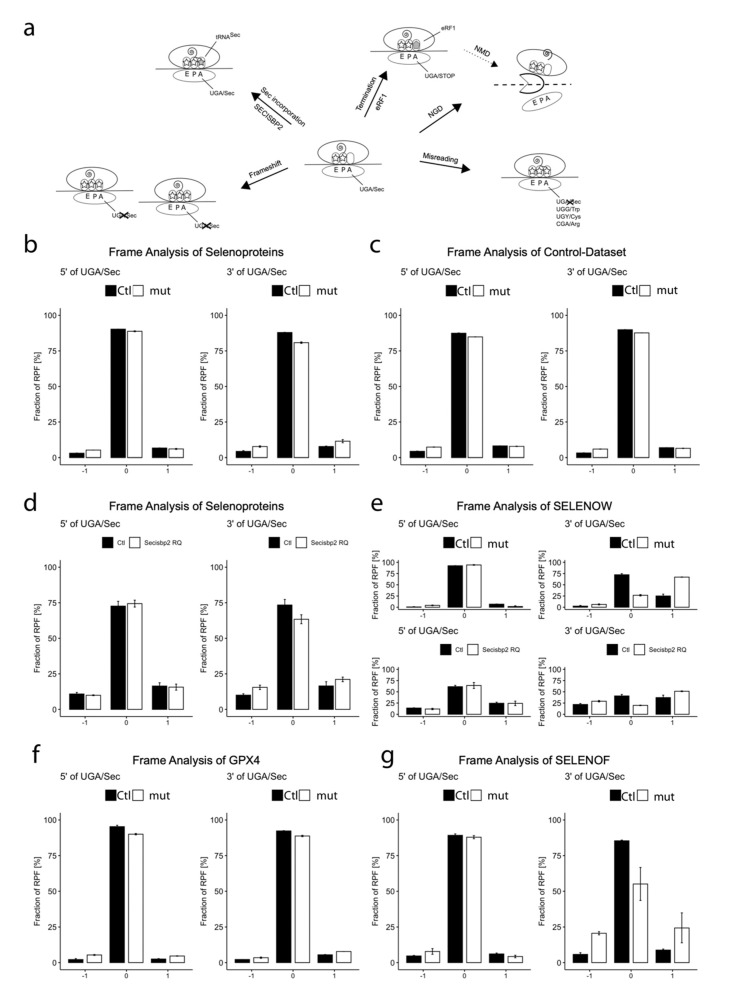
Reading frame analysis of selenoproteins. (**a**) Possible outcomes when a translating ribosome encounters a UGA/Sec codon. If Sec-tRNA^Sec^ is not readily available, termination, mRNA degradation, misreading or frameshifting may occur. The choice is influenced by trans-acting factors such as *SECISBP2*, possibly others, and by the context of primary or secondary (i.e., selenocysteine redefinition elements) RNA structure. In addition, incomplete modification of tRNA^Sec^ may also disfavor efficient Sec incorporation depending on mRNA context. (b) The fraction of RFPs (%) in frame –1, 0 and +1 5′ (left) and 3′ (right) of the UGA/Sec codon is represented for control and *SECISBP2*-mutant HAP1 cells. (**c**) The fraction of RFPs (%) in frames –1, 0 and +1 5′ (left) and 3′ (right) for a control dataset, consisting of the whole transcriptome except for the selenoproteins. A hypothetical UGA/Sec was set at the center of every transcript. (**d**) The fraction of RFPs (%) in frames –1, 0 and +1 5′ (left) and 3′ (right) of the UGA/Sec codon is represented for the cortex of control and neuron-specific *Secisbp2^R543Q/fl^* mice. (**e**) The fraction of RFPs (%) of SELENOW in frames –1, 0 and +1 5′ (left) and 3′ (right) of the UGA/Sec codon is represented for control and *SECISBP2*-mutant HAP1 cells (upper panel) and for cortex of control and neuron-specific *Secisbp2^R543Q/fl^* mice (lower panel). (**f**,**g**) The fraction of RFPs (%) in frames –1, 0 and +1 5′ (left) and 3′ (right) of the UGA/Sec codon is represented for GPX4 (**f**) and SELENOF (**g**) in control and *SECISBP2*-mutant HAP1 cells.

At this point, we have to consider all the processes that might occur once the ribosome encounters a UGA/Sec codon (Figure 6a). The ideal situation, clearly, is elongation upon selection of Sec-tRNA^Sec^. This process is enhanced by the presence of *SECISBP2* but occurs even with a mutated *SECISBP2*, albeit at a lower efficiency, which apparently depends on the transcript. Transfer tRNA^Sec^ is rare compared with other canonical tRNAs [49]. Accordingly, termination, the competing process, is likely preferred, if *SECISBP2* cannot support elongation. There is reason to believe that codon context around the UGA/Sec codon disfavors termination in several selenoproteins [50]. Otherwise, the termination process should prevail, and translation of a selenoprotein such as SELENOP, that contains, depending on species, 10-17 Sec would be practically impossible [51]. If the UGA/Sec codon is read as a termination codon, termination will occur, and further signals are integrated to decide whether the mRNA is degraded involving NMD. *GPX1* is a well-established target of NMD [52]. This process is apparently repressed by *SECISBP2*, since Ribo-Seq analysis revealed stalling of ribosomes close to UGA in tRNA^Sec^-deficient mouse liver, where *SECISBP2* was expressed [26]. Impaired *SECISBP2* function, in turn, leads to reduced *GPX1* mRNA levels, eliminating potential evidence of ribosome stalling at the UGA. If termination would not occur, but neither elongation, no-Go decay (NGD) would be a possibility to release the stalled ribosome [53]. An additional possibility that has been observed by amino acid analysis is misreading of the UGA codon by a near-cognate codon UGG/Trp, UGY/Cys, or CGA/Arg [48].

### 3.7. Evidence for Frameshifting at UGA Codons in Selenoprotein Translation

We wondered whether there exists still another possibility to escape ribosomal stalling at the UGA/Sec, i.e., ribosomal frameshifting. Such an event can be readily detected using high-resolution Ribo-Seq. In order to uncover frameshifting events at the UGA/Sec codons, we determined the fraction of selenoprotein RPF in frame 0 on both sides of the UGA/Sec. In the case of frameshifting, the fraction of RPF in frame 0 should decrease 3′ of the UGA/Sec codon. When summed over all selenoprotein RPF in Ctl HAP1 cells, the fraction of RPF in frame 0 3′ of the UGA/Sec was not different from the fraction of RPF in frame 0 5′ of the UGA (Figure 6b). In contrast, in *SECISBP2*-mutant cells, the fraction of RPF in frame 0 dropped 3′ of the UGA/Sec (Figure 6b). Accordingly, 3′ RPF in frames +1 and −1 increased. In order to show that mutant *SECISBP2* does not trigger generalized frameshifting, we analyzed the whole transcriptome excluding selenoprotein transcripts. We placed an imaginary UGA/Sec codon at the center of each transcript and compared the C-terminal RPFs with the N-terminal RFPs (Figure 6c). We did not detect higher amounts of out-of-frame RFPs for in this non-selenoprotein control dataset. The finding of frameshifting at the UGA/Sec did not entirely surprise us, because we had made a similar observation in a recent study applying Ribo-Seq in neuron-specific *Secisbp2^R543Q^*-mutant mice [27]. Because of the limitations of the previous study (more heterogenous cell material in brain), we had not been confident enough to highlight this finding. After we replicated this finding in an independent dataset with different procedures and in a genetically homogenous human cell population, we can now show these results with confidence (Figure 6d). 

**Figure A4:**
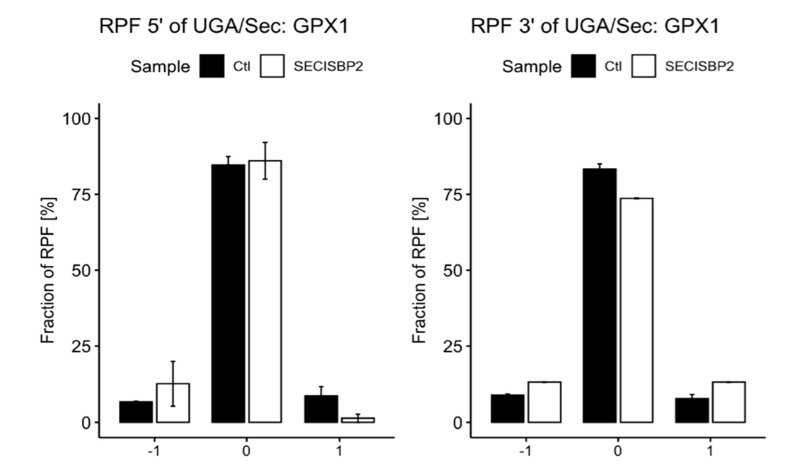
Frameshifting at the UGA/Sec codon in *GPX1* occurs in HAP1 *SECISBP2*-mutant cells. While the fraction of RPFs in frame 0 decreases 3′ of the UGA/Sec, the fraction of frame +1 RPFs increases.

A more detailed analysis revealed that the largest contribution to frameshifting came from only few selenoproteins, i.e., frameshifting at the UGA/Sec is a gene-specific event. The selenoprotein mRNA with the greatest effect in both the human cells and the *SECISBP2*^R543Q^ mouse model was *SELENOW* (Figure 6e). Here, less than 30% of RPF 3′ of the UGA/Sec remained in frame 0 in *SECISBP2*-mutant cells. Remarkably, frameshifting occurred also in the control cells, but at a much smaller rate. The most abundantly expressed selenoprotein, *GPX1*, also contributed to frameshifting after the UGA/Sec codon (Figure A4), while *GPX4* did not show any indication of frameshifting (Figure 6f). In addition, pronounced frameshifting occurred in *SELENOF* but to a lower extent than in *SELENOW* (Figure 6g). Thus, two *SECISBP2*-mutant models (*SECISBP2*^R543Q^ and the *SECISBP2*^Δ^^*623−630,D631N*^) consistently demonstrated frameshifting events at the UGA/Sec codon in the same set of selenoproteins.

## 4. Discussion

### 4.1. Mutated SECISBP2 and UGA/Sec Re-Coding

Our cell model carries an in-frame deletion of eight amino acids (positions 623–630) and the missense mutation Asp631Asn in *SECISBP2*, which is caused by the skipping of mutated exon 13 (Figure 1b and Figure A1). These amino acids mark the conserved N-terminus of the L7Ae RNA-binding domain, which interacts with the SECIS element. The paralogue *SECISBP2L* carries only two conservative amino acid exchanges among these same nine positions (Figure A1b). In a recent cryo-EM structure of a ribosome decoding the UGA/Sec codon, these nine amino acids are not well-resolved but may interact with either SECIS and/or the ribosome [13]. ΔUREs in *SECISBP2*-mutant HAP cells are generally more similar to ΔURE in *Secisbp2*-knockout liver than ΔURE in *Secisbp2^R543Q^*-mutant brain (Table A1) [26,27]. Considering the impaired ^75^Se-metabolic labeling of selenoproteins and other results in *SECISBP2*-mutant HAP cells, we deem the mutant *SECISBP2* functionally impaired and close to a functional null. Our data are consistent with a recent report, in which GPX4 and TXNRD1 were still expressed in diffuse large B-cell lymphoma cell lines apparently deficient in *SECISBP2* immunostaining, although it was not further studied what kind of mutations may have caused the lack of *SECISBP2* protein [54]. Residual expression of GPX4 and TXNRD1 was observed in patients carrying mutations in *SECISBP2* [15]. These studies tested a more limited set of selenoproteins compared with our studies. For example, the homozygous R540Q mutation in *SECISBP2* led to significantly reduced levels of GPX1 and DIO2 activity in patient fibroblasts, while GPX3 was less affected in patient serum [14]. These observations opened the possibility that the residual selenoprotein expression in *SECISBP2*-deficient models may be due to the activity of *SECISBP2L*. Accordingly, a recent study in zebrafish suggested that *SECISBP2L* may be involved in the translation of at least a few selenoproteins in vivo [22]. In addition, inactivation of *Secisbp2L* in mouse oligodendrocyte precursor cells abrogated expression of DIO2 [23]. Other selenoproteins, however, were not studied in this work. While *SECISBP2L* is expressed in HAP1 cells (and unchanged in *SECISBP2*-mutant cells, not shown), we cannot comment on the role of *SECISBP2L* in our model because *DIO2* is, according to Ribo-Seq, not translated at all in HAP1 cells.

### 4.2. SECISBP2 Opposes Termination

In mouse models of low selenium availability or mutation of tRNA^Sec^, several selenoproteins show ribosomal pausing at or upstream of the UGA/Sec codon [26,28]. Pausing was not observed in the same transcripts when *Secisbp2* was inactivated [26]. This observation implicates that *SECISBP2* can delay for a significant time premature termination when bound to a ribosome with a UGA/Sec codon in the A-site. 

### 4.3. Does SECISBP2 Help Recruit Sec-tRNA^Sec^:EEFSEC?

All the measures to estimate read-through of UGA/Sec in individual transcripts via Ribo-Seq are based on the density of RPF 3′ of the UGA/Sec and, depending on the method, are influenced by RPF density 5′ of the UGA or by mRNA instability. We wanted to harness the full power of Ribo-Seq and analyzed our Ribo-Seq data in this study at codon resolution around the UGA/Sec codon. Ribosomes reading the UGA/Sec codons have the tRNA^Sec^ positioned in the A-site. Such RPF are characterized by their length of 28/29 nucleotides (bRPF), and the codon adjacent to the A-site is 15 nucleotides from their 5′ terminus (see Methods). Clearly, selenoprotein transcripts with reduced UGA/Sec read-through are expected to have fewer of such RPFs. Likewise, if recruitment of Sec-tRNA^Sec^ was impaired, one would expect an increased number of RPFs with UGA/Sec adjacent to an empty A-site. Such RPF have a size of 21/22 nucleotides. We believe this type of RPF is analyzed for the first time in the present study. *SELENOH* and *SELENOO* are the transcripts with the most abundant RPF coverage on the UGA/Sec codon in our HAP1 cell model and are both resistant to NMD. Thus, RPF density is not confounded by changing levels of mRNA. In both cases, the increase in sRPF (UGA/Sec in A-site, no aa-tRNA recruited) is clearly seen in *SECISBP2*-mutant cells. We conclude from this data that such an observation is compatible with a function of *SECISBP2* in recruitment of EEFSEC:Sec-tRNA^Sec^ to the ribosomal A-site—possibly by stabilizing a conformation of the SECIS that can interact with EEFSEC [13].

### 4.4. pUGA as a Measure for UGA/Sec Redefinition Efficiency

Next, the most direct way to look at Sec incorporation using Ribo-Seq is to assess the density of RPFs with UGA/Sec positioned in the P-site (pUGA). Such RPF directly reflect successful elongation through UGA. This measure can be calculated irrespective of the occupancy of the A-site 3′ from the UGA/Sec. Thus, by pooling sRPF and bRPF, the data base is improved on the level of individual transcripts. pUGA nicely correlates with 3′RPM but can be calculated also for selenoproteins carrying the UGA/Sec codon close to the termination codon (Figure 3e). Hence, we can finally provide a measure of UGA/Sec read-through for all selenoproteins, including those with the UGA/Sec close to the C-terminus. For such detailed analysis, considerable sequencing depth is required, and studies on selenoproteins with low expression levels would profit from some sort of enrichment for selenoprotein mRNAs before performing Ribo-Seq. 

### 4.5. Frameshifting, a New Process at UGA/Sec

Translational frameshifting has, to our knowledge, not been explored in the context of canonical selenoproteins. There is, however, a known instant where a synthetic UAG/Sec codon in GPX4 recombinantly expressed in an *RF1*-mutant *E. coli* was skipped in a +3 frameshifting event [55]. This may indicate that ribosomes apparently try to avoid termination in a codon context not optimized for termination [50]. Often, translational frameshifting is stimulated by a “hungry codon” in the context or of a “slippery site” [56,57,58]. Inspection of our Ribo-Seq data finds evidence of increased frameshifting in selenoproteins in HAP1 cells expressing *SECISBP2*^D623−630, Δ631N^. The mRNA with the biggest effect is *SELENOW* but also *GPX1* and *SELENOF* showed a reduced fraction of RPF in frame 0 3′ from the UGA/Sec codon. We failed to pinpoint a specific “slippery” sequence in these selenoproteins, but it is obvious that UGA/Sec would qualify as a “hungry codon”, since Sec is by far the rarest proteinogenic amino acid. In the case of the Ty3 retrotransposon in yeast, +1 frameshifting in the P-site has been shown at a GCG/Ala codon followed by the “hungry” AGU/Ser codon without the need of a “slippery site” [57]. In *SELENOW*, the P-site codon GCU also encodes Ala. It is interesting that even in the presence of functional *SECISBP2*, there seems to be increased +1 frameshifting in *SELENOW*. We found the same increased frameshifting 3′ from the UGA/Sec in *Selenow* and *Selenof* in mice expressing the *SECISBP2*^R543Q^ mutant [27]. The sequencing depth in the older experiment was lower than in our present experiment; in light of the new data, however, we have convinced ourselves. In addition, the reader may have noticed the termination products in the Western Blot of SELENOF in Figure 1e. Simple termination at the UGA/Sec codon would not be able to explain several truncated protein bands. 

### 4.6. Potential Technical Limitations

Considering that our present experiment assigns more than 85% of all RPF to reading frame 0 in the control HAP1 cells, we consider this Ribo-Seq experiment to be of high quality. The RPF assigned to other reading frames are the result of incomplete nuclease digestion at the 5′ end of the RPF and merely reflects a technical limitation. If a clear increase in RPF outside frame 0 is observed in only specific selenoproteins and only 3′ of the UGA/Sec, we take this as evidence for frameshifting. Clearly, future studies should investigate in detail the codon context and conditions in favor of frameshifting.

When we consider pUGA, we assume that the tRNA occupying the P-site is Sec. However, our Ribo-Seq experiment cannot inform us about the identity of this tRNA. In addition to elongation with Sec, there is the possibility of misreading with a near-cognate tRNA. Mis-incorporation of Trp, Arg, and Cys at UGA/Sec have been demonstrated upon aminoglycoside treatment [48]. Thus, there is the theoretical possibility that we overestimate pUGA if near-cognate selection occurs at significant frequency. 

## Figures and Tables

**Figure A3:**
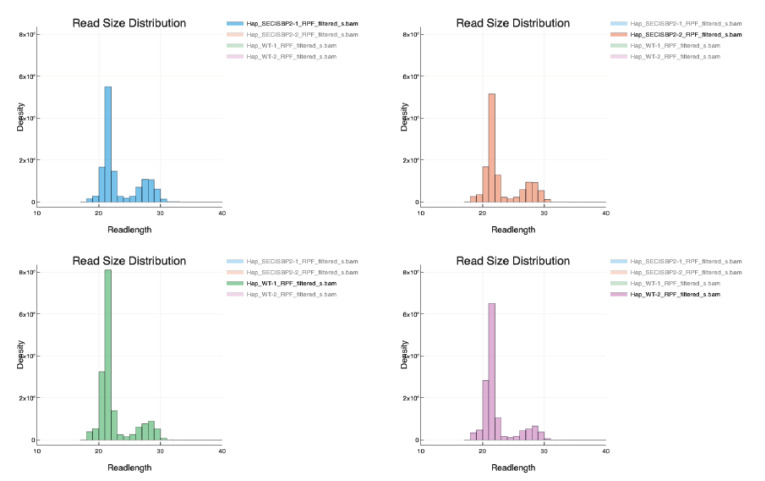
Size distribution of reads mapping to coding sequences of transcripts located in the cytoplasm and expressed in HAP1 cells. Reads around 21 nucleotides length represent “small” ribosomal footprints (sRPF, A-site not occupied) and reads of 28 nucleotides length represent “big” RPFs (A-site occupied). For pUGA and related measures, only reads of 20 + 21 and 28 + 29 nucleotides were selected, since in these RPFs, the first base of the P-site is 12 nucleotides from the 5′ end. These species are also the most abundant species in their respective peaks.

## Data Availability

Raw sequence data and raw counts were deposited at the NCBI GEO repository (https://www.ncbi.nlm.nih.gov/geo/), entry GSE145465.

## Supplementary Materials

The following supporting information can be downloaded at: https://www.mdpi.com/article/10.3390/biom12101504/s1, Table S1. PCR primers and conditions: Table S2. Antibodies.

## Appendix A

**Table A1:** Effects of mutations in *SECISBP2* genes on UGA re-coding efficiency (URE) of selenoproteins.

Selenoprotein	URE Wild-Type	URE Mutant	ΔURE ± SD
GPX1	0.30	0.35	1.12 ± 0.02 ^a^
	1.04	1.13	1.08 ± 0.29 ^b^
	1.84	1.82	0.99 ± 0.06 ^c^
GPX4	0.47	0.11	0.24 ± 0.01 ^a^
	2.07 0.69	0.62 0.31	0.30 ± 0.02 ^b^ 0.45 ± 0.01 ^c^
SELENOF	1.41	0.40	0.29 ± 0.01 ^a^
	0.25	0.14	0.58 ± 0.17 ^b^
	0.34	0.04	0.12 ± 0.01 ^c^
SELENOH	0.49	0.45	0.93 ± 0.04 ^a^
	0.59	0.90	1.51 ± 0.03 ^b^
	0.40	0.22	0.55 ± 0.00 ^c^
SELENOT	0.19	0.23	1.19 ± 0.08 ^a^
	1.39	1.56	1.12 ± 0.15 ^b^
	2.00	0.60	0.30 ± 0.05 ^c^
SELENOW	0.50	0.40	0.80 ± 0.27 ^a^
	0.28	0.37	1.29 ± 0.02 ^b^
	2.59	1.67	0.64 ± 0.13 ^c^
SEPHS2	2.69	0.87	0.32 ± 0.01 ^a^
	0.62	0.48	0.77 ± 0.06 ^b^
	2.02	0.52	0.26 ± 0.01 ^c^

^a^ *Secisbp2-knockout,* Fradejas-Villar et al. [26]; ^b^ *Secisbp2^R543Q,^* Zhao et al., [27]; ^c^ *SECISBP2*^Δ*623−630,D631N*^, this work. ΔURE = (URE mut/URE Ctl). SD, standard deviation. *SECISBP2*^Δ*623−630,D631N*^ is more similar to *Secisbp2*-knockout than *Secisbp2^R543Q^*.
