# Supplementary material for: High-Resolution Ribosome Profiling Reveals Gene-Specific Details of UGA Re-Coding in Selenoprotein Biosynthesis
Source: Biomolecules. 2022 Oct 17;12(10):1504. doi: 10.3390/biom12101504 (PMC9599142; doi:10.3390/biom12101504)
Supplement: Supplementary file 1 [file biomolecules-12-01504-s001.zip › biomolecules-1922316-supplementary.pdf]

Table S1. PCR primers and conditions to amplify genomic and cDNA from control and *SECISBP2* KO cells.

Primer Name	Primer Purpose	Sequence
SECISBP2_ Fw	gDNA PCR	5′-TATCTGATGATGCCTTTTTCCTCCA-3′
SECISBP2_Rv	gDNA PCR, sequencing	5′-ACAAGAACCTCAGTAGAAGCAGAAT-3′
hSECISBP2_E12_Fw	cDNA PCR and sequencing	5′-TGGTGATGACCAGTTTCCCG-3′
hSECISBP2_E14_Rv	cDNA PCR	5′-CCCTCAACCCCAACACAAGT-3′

PCR conditions for amplification and Sanger sequencing of PCR products. For genomic DNA: 35cycles, $T_{annealing}$ 58 °C, $t_{extension}$ 1 min. Conditions for amplification of cDNA: 35 cycles, $T_{annealing}$ 60 °C, $t_{extension}$ 30s.

Table S2. Antibodies used in this study.

Antibody	Host Species	Dilution	Manufacturer	Catalogue Number
SECISBP2	Rabbit	1:1000	Abcam	ab210791
Beta-ACTIN	Mouse	1:25000	Sigma Aldrich	A3854
GPX1	Rabbit	1:1000	Abcam	ab22604
GPX4	Rabbit	1:1000	Abcam	ab125066
SELENOT	Rabbit	1:250	Sigma	HPA039780
SELENOM	Rabbit	1:1000	Sigma	HPA019601
TXNRD1	Mouse	1:1000	Abcam	ab16847
SELENOH	Rabbit	1:500	Santa Cruz	SC382288
SELENOK	Mouse	1:500	Sigma	HPA008196
SELENOS	Rabbit	1:1000	Sigma	HPA010025
SELENOF	Rabbit	1:5000	Abcam	ab124840
TXNRD2	Rabbit	1:1000	Atlas	HPA003323
HRP goat anti mouse	Goat	1:1000	Jackson Immunotech (Dianova)	115-035-003
HRP goat anti rabbit	Goat	1:1000	Jackson Immunotech (Dianova)	111-035-003
